# Analysis of Plasmonic Sensors Performance Realized by Exploiting Different UV-Cured Optical Adhesives Combined with Plastic Optical Fibers

**DOI:** 10.3390/s23136182

**Published:** 2023-07-06

**Authors:** Francesco Arcadio, Chiara Marzano, Domenico Del Prete, Luigi Zeni, Nunzio Cennamo

**Affiliations:** Department of Engineering, University of Campania Luigi Vanvitelli, Via Roma 29, 81031 Aversa, Italy; francesco.arcadio@unicampania.it (F.A.); chiara.marzano@studenti.unicampania.it (C.M.); domenico.delprete@unicampania.it (D.D.P.); luigi.zeni@unicampania.it (L.Z.)

**Keywords:** surface plasmon resonance (SPR), plastic optical fibers (POFs), optical sensors, polymer-based optical waveguides, UV-cured optical adhesives, 3D-printed sensor chips

## Abstract

Polymer-based surface plasmon resonance (SPR) sensors can be used to realize simple, small-size, disposable, and low-cost biosensors for application in several fields, e.g., healthcare. The performance of SPR sensors based on optical waveguides can be changed by tuning several parameters, such as the dimensions and the shape of the waveguides, the refractive index of the core, and the metal nanofilms used to excite the SPR phenomenon. In this work, in order to develop, experimentally test, and compare several polymer-based plasmonic sensors, realized by using waveguides with different core refractive indices, optical adhesives and 3D printed blocks with a trench inside have been used. In particular, the sensors are realized by filling the blocks’ trenches (with two plastic optical fibers located at the end of these) with different UV-cured optical adhesives and then covering them with the same bilayer to excite the SPR phenomenon. The developed SPR sensors have been characterized by numerical and experimental results. Finally, in order to propose photonic solutions for healthcare, a comparative analysis has been reported to choose the best sensor configuration useful for developing low-cost biosensors.

## 1. Introduction

The surface plasmon resonance (SPR) phenomenon is triggered by the interaction between an incident light and free electrons at the surface of an ultrathin noble metal film (e.g., gold, silver, etc.). In particular, when an incident light at a metal nanofilm in contact with a dielectric medium is such as to satisfy certain resonance conditions, a plasmonic phenomenon is excited along the metal–dielectric interface. The SPR technique is highly sensitive to changes in the refractive index of the encompassing dielectric medium that is in contact to the metal film [1,2]. During the last few decades, SPR sensors have been widely utilized in several research areas. More specifically, concerning the biochemical sensing field, plasmonic sensors provide a number of advantages over conventional biochemical assays, including high sensitivity, label-free detection, real-time monitoring, and the possibility to evaluate binding kinetics [3,4,5,6,7,8].

Despite traditional plasmonic sensors are based on prism coupling and Kretschmann configuration that typically require bulky experimental setups [9,10,11], currently, the SPR technique is frequently coupled with optical waveguides to reap the advantages related to the use of optical components, such as tiny dimensions, immunity to electromagnetic interference, capability of remote sensing, and so on. As an example, several SPR configurations based on both glass and plastic optical fibers (POFs) have been presented in the literature [12,13,14,15]. 

In general, within the class of plasmonic sensors based on optical waveguides, polymeric-based SPR sensors are typically preferred over glass-based sensors since they denote numerous benefits including flexibility, easy handling, low-cost, and an easy manufacturing process. Along this line, SPR optical sensors based on plastic substrates have been presented to detect specific substances, by combining the sensitive areas with specific receptors either biological (e.g., antibodies, aptamers, enzymes, etc.) [16,17,18] or synthetic (such as molecularly imprinted polymers (MIPs)) [19,20]. 

Recently, the rapid growth of 3D-printing technologies has remarkably boosted the development of cutting-edge sensors [21,22]. Above all, the possibility to achieve more complicated geometries than simple cylindrical fibers is one of the most significant benefits of using 3D printing techniques. In this regard, Hinman et al. [23] proposed an SPR biosensing approach based on Kretschmann-like configuration by exploiting the 3D printing of equilateral prisms by using a commercial photocurable resin. Alternatively, other techniques call for the use of pricey resin [24], which raises the overall cost of the conceived sensor. Furthermore, Cennamo et al. recently exploited a low-cost approach based on 3D inkjet-printing technology combined with photoactive resins to build innovative SPR sensors [25].

In this work, by using a similar approach described in [25], a comparative analysis with regard to three different 3D-printed plasmonic configurations based on photocurable resins was performed. More specifically, the resins, acting as the waveguides’ core and jetted into a 3D-printed support, had a variable refractive index in order to study the influence of this parameter above the plasmonic performances. Firstly, numerical results were achieved to extrapolate a trend above the plasmonic response of the proposed sensor configurations. Then, the three configurations having a core refractive index equal to 1.48, 1.50, and 1.54, respectively, were fabricated and optically characterized by using water–glycerin solutions. 

The proposed sensitive approach offers the possibility to achieve significant benefits by using 3D printing techniques. In particular, the shape of the 3D-printed trench could be modified to change the sensor performance and capabilities in several application fields. For instance, by changing the cross-section dimension/shape of the trench via an optimization analysis obtained by fixing the refractive indices, we could improve the performance; however, by changing the shape of the trench along the light propagation direction (top-view section), e.g., with a V-shape or U-shape geometry, the sensor chip could be monitored by a connection with a Light Emission Diode (LED) and a Charge Coupled Device (CCD) of smartphones to realize simple Internet of Things (IoT) sensor systems.

## 2. Theoretical Background

The sensing principle relies on the SPR phenomenon excited at the boundary between a gold nanofilm and a dielectric medium (liquid solution). More specifically, when a water solution is present on the gold surface (plasmonic sensitive surface), the SPR phenomenon takes place and a dip in the normalized transmitted spectrum occurs. The resonance wavelength and the shape of the dip are function of the refractive index of the solution (dielectric medium) and of the mode profile of the light propagating into the core of the multimode POF [26,27], as reported in the following Equations (1) and (2):

(1) K0ncsinθ=K0(εmrns2εmr+ns2)12; K0=2πλ

(2) Ptrans=∫θcrπ2ReNref(θ)I(θ)dθ∫θcrπ2I(θ)dθ 

In Equation (1) (SPR condition for each single mode), the term on the left-hand side is the propagation constant (*K_inc_*) of the evanescent wave, where *n_c_* is the refractive index of the device used to couple the light (such as a prism or the core of optical fibers) and *θ* is the light incident angle; the right-hand term is the surface plasmon wave propagation constant (*K_SP_*), where *ε_mr_* is the real part of the metal permittivity and *n_s_* is the refractive index of the dielectric medium [26,27]. 

Equation (2) shows the normalized transmitted power (*P_trans_*) of an SPR sensor realized in POFs (multimode optical fibers), where *θ_cr_* is the critical angle, *R_e_* is the reflectance, *I*(*θ*) is the angular intensity distribution related to the employed light source, and *N_ref_*(*θ*) corresponds to the number of multiple reflections and it is related to the plasmonic sensing area length and the fiber core diameter [27].

Therefore, the sensor’s sensitivity (*S*) can be defined by calculating the shift in resonance wavelength per unit change in refractive index (nm/RIU). More in detail, the sensitivity of the sensor can be defined as follows [26,27]:

(3) $$S={\left(\frac{\delta \lambda_{res}}{\delta n_{s}}\right)}_{n_{s}}\;\;\;\;\;\left[\;\frac{\mathrm{nm}}{\mathrm{RIU}}\;\right]$$

where *δλ_res_* is the resonance wavelength shifts due to a variation in the refractive index of the sensing layer of *δn_s_*.

The sensor’s resolution (Δ*n*) can be defined as the minimum amount of the refractive index variation measurable by the sensor system. So, it can be calculated as follows [26,27]:

(4) Δn=δnsδλres δM=1S δM[RIU]

where δ*M* is standard deviation of the resonance wavelength and it depends on the spectral resolution of the used spectrometer.

**Figure 1:**
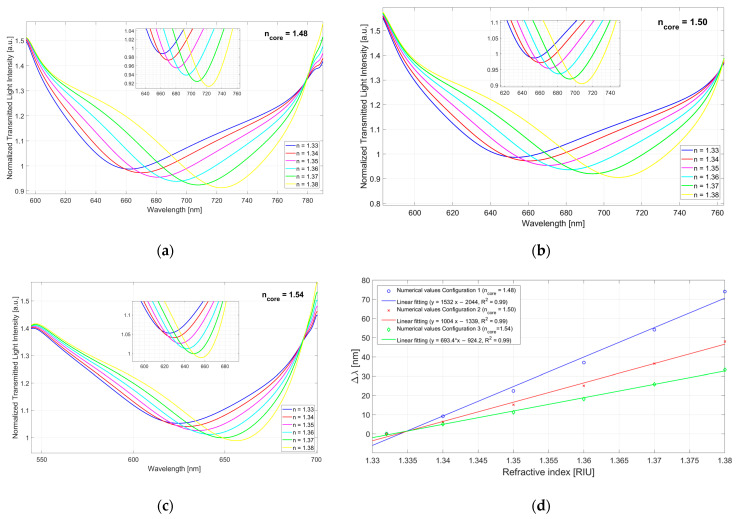
Numerical results obtained by three configurations with different core refractive index values (*n_core_*). (**a**) Numerical SPR spectra relative to Configuration 1 (*n_core_* = 1.48). (**b**) Numerical SPR spectra relative to Configuration 2 (*n_core_* = 1.50). (**c**) Numerical SPR spectra relative to Configuration 3 (*n_core_* = 1.54). (**d**) Numerical variation in resonance wavelength (∆λ), calculated with respect to the value 1.332, versus external medium refractive index (*n_s_*), together with the linear fitting of the numerical data, for the three sensor configurations: Configuration 1 (*n_core_* = 1.48), Configuration 2 (*n_core_* = 1.50), and Configuration 3 (*n_core_* = 1.54).

By using Equations (1) and (2), numerical results have been achieved using MATLAB software, as described in [27]. The latter is based on a multilayer approach and takes advantage of the transfer matrix formalism to simulate the SPR sensor response [27]. The refractive index of the cladding is fixed to the value of 1.475, the one of the buffer layer (placed over the core and under the gold film) is 1.61, while the refractive index of the core is *n_core_* = 1.48 (Configuration 1), *n_core_* = 1.50 (Configuration 2), and *n_core_* = 1.54 (Configuration 3); on the other hand, the values of the simulated multilayer waveguide’s thicknesses are: 1 mm for the core, 1.5 µm for the buffer layer, and 60 nm for the gold. Therefore, Figure 1 shows the numerical results obtained by using three refractive index values of the waveguide’s core (*n_core_*), by fixing the other simulated multilayer waveguide’s parameters (dimensions and refractive index values of the other layers), and by changing the refractive index of the dielectric medium in contact with the gold surface (*n_s_*). More specifically, Figure 1a reports the numerical SPR spectra relative to *n_core_* = 1.48 (Configuration 1), Figure 1b is relative to *n_core_* = 1.50 (Configuration 2), whereas Figure 1c is relative to *n_core_* = 1.54 (Configuration 3). Finally, Figure 1d shows the numerical resonance shift (Δ*λ*), calculated with respect to the 1.332 value, versus the solution’s refractive index, for the three different sensor configurations, together with the linear fitting of the numerical data.

## 3. SPR Sensor Chips and Setup

### 3.1. Manufacturing Steps of the Sensor Systems

**Figure 2:**
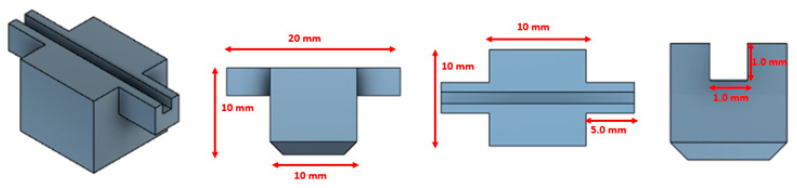
Schematic cross sections of the 3D-printed support used to create the channel hosting UV-sensitive optical adhesives.

Three different sensor configurations were fabricated by using the same geometry and changing the optical characteristics of the waveguide core. More specifically, each configuration was created through a 3D-printed support which includes a slot that was filled with three different UV-cured optical adhesives having different refractive indices (*n*) equal to 1.48, 1.50, and 1.54, respectively. Therefore, we have fixed the waveguide’s dimensions and the plasmonic multilayer (gold film and S1813 layer) in order to compare the sensors’ performances at the variation in the refractive index of the core (UV-cured optical adhesive). In particular, we have used a fixed size of 1 mm × 1 mm trench (see Figure 2) to realize optical waveguides similar to those based on POFs with 1 mm in diameter [26] but with different refractive indices.

More in detail, three UV photopolymer optical adhesives (Norland, Jamesburg, NJ, USA) having different refractive indices were used to create the core of the waveguide useful to achieve the plasmonic sensor. Three configurations were thus obtained: the first having a core refractive index equal to 1.48 (NOA 148), the second having a refractive index equal to 1.50 (NOA 78), and the third with a refractive index equal to 1.54 (NOA 68). These NOA optical adhesives present the same spectral transmission value of 100% in the range of interest from 450 nm to 800 nm (other characteristics are reported in the relative NOA’s data sheet).

This simple approach made it possible to carry out a comparative analysis of the plasmonic resonance conditions excited by the waveguides with the same geometry, reported in Figure 2, by varying only the refractive index of the core.

At first, it was necessary to create a support from which to obtain the three different configurations to be studied; the latter was designed by using a commercial software (Autodesk Fusion 360), and Figure 2, reports schematic cross sections which highlight the designed support dimensions (1 mm × 1 mm). As reported in Figure 2, the printed trench is 2 cm long, and it is used to fill it with optical adhesive and to place the POFs at its end. It should be underlined that the designed slot area is 1 mm^2^ in order to compare the performance of the proposed sensor configurations with respect to other polymer-based SPR sensors, such as [17,19,25,26].

Next, the above-mentioned support was printed through a 3D printer (Photon Mono X UV Resin SLA 3D Printer, Anycubic^®^, Shenzhen, China), which presents an 8.9-inch 4K LCD with 3840 × 2400 pixels that corresponds at an XY resolution of 50 μm. For printing, a gray UV-sensitive resin was used, characterized by having high precision and short times for photopolymerization. The refractive index of this resin (the cladding of the waveguide) is about 1.475 in the visible range. 

Once the supports were ready, a first jetting of UV-cured optical adhesive was carried out inside the prepared slot. Before the photopolymerization of the optical adhesive took place, two plastic fiber optic (POF) patches (1 mm total diameter) were inserted at the two ends to launch the input light and collect it at the exit. At this point, the three platforms were cured for approximately 10 min under a lamp bulb with UVA emission at 365 nm. Following the UV-curing of the first layer of optical adhesive, it was found that the UV-cured optical adhesive exhibited significant shrinkage, which reduced the size of the core compared to that initially designed. The core diameter is strictly related to the waveguide multimodality which plays a key aspect when dealing with plasmonic sensors based on optical waveguides [27,28]. Standing this, a second jetting of the optical adhesive was performed to obtain the designed 1 mm × 1 mm waveguide. After having polymerized the three configurations for another 10 min under a lamp bulb with UVA emission, they were subjected to a polishing process using first a paper having grains of 5 µm and subsequently of 1 µm [26]. 

**Figure 3:**
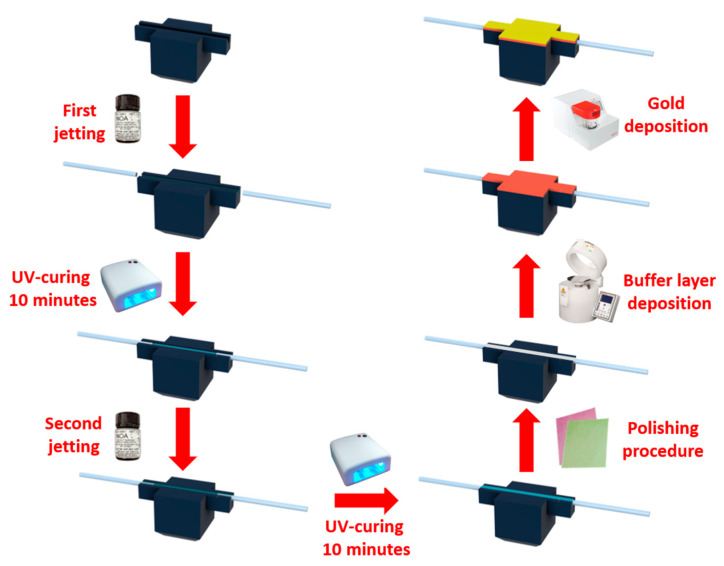
Schematization of the fabrication steps of the plasmonic sensor chips based on cured optical adhesives and POFs.

We have used the polishing process on the planar surface in order to obtain the same roughness on several sensor configurations (similar to [26]). This aspect is necessary to achieve by spinning process the same thickness of the S1813 buffer layer on the compared sensor configurations. In fact, a photoresist buffer layer (Microposit S1813, Allresist GmbH, Strausberg, Germany) of about 1.5 µm in thickness was deposited by a spin coater (dropping and spinning at 6000 rpm, by a Spin coater, model WS-650MZ-23NPPB, manufactured by Laurell Technologies Corporation, Lansdale, PA, USA) [26]. This micrometric layer provides two different benefits, first to improve the plasmonic performance of the waveguide and second to improve the adhesion between the optical resin and the nanometric gold layer which will be deposited in the next fabrication step [26]. To guarantee the fulfillment of the surface plasmon resonance condition, it was indeed necessary to deposit a nanometric film, particularly about 60 nm of gold, on the sensitive area by using a sputter coater machine (Safematic CCU-010, Zizers, Switzerland). Figure 3 shows an outline the various construction steps adopted for the realization of the three platforms based on 3D printing and optical adhesives.

**Figure 4:**
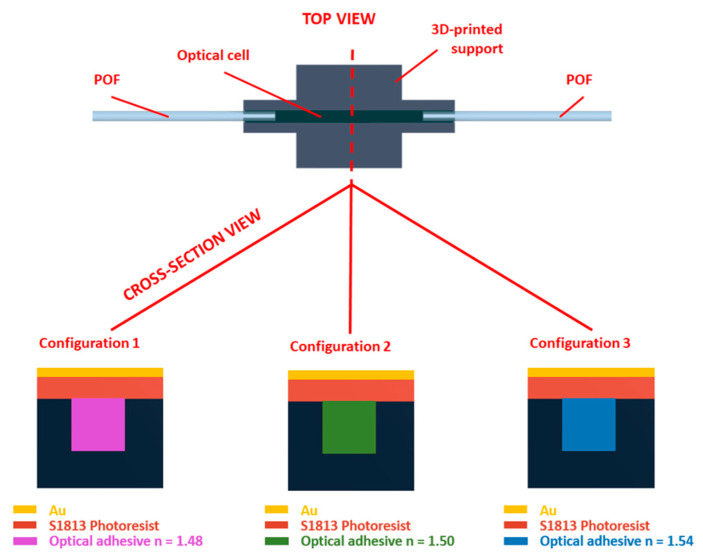
Schematic views of the proposed SPR configurations based on different waveguide’s core refractive index.

Once the following steps were carried out, the three configurations under analysis were obtained. Figure 4 reports a schematic top view and the respective sectional views for each configuration named as follows: “Configuration 1”, with a core index equal to 1.48; “Configuration 2”, having a core whose refractive index is equal to 1.50; and, finally, “Configuration 3”, with a core refractive index equal to 1.54.

### 3.2. Experimental Setup

**Figure 5:**
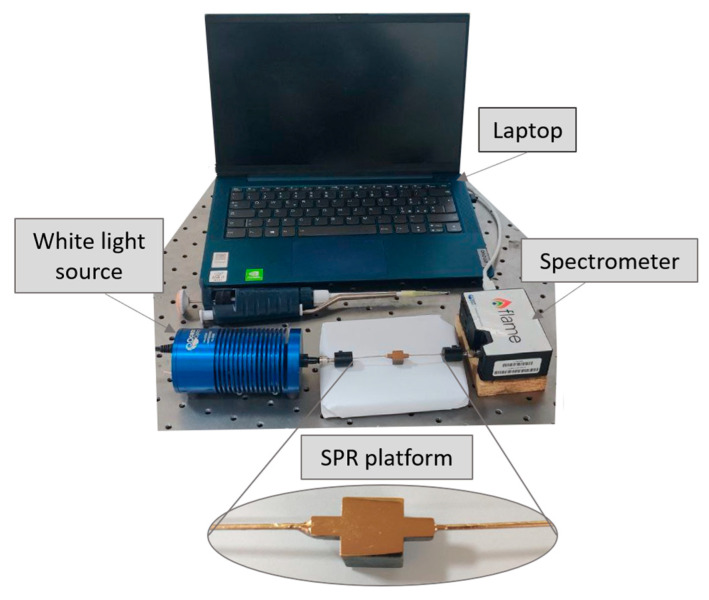
Actual image of the experimental setup used to test the proposed SPR chip configurations.

In order to characterize the three configurations from an optical point of view, a simple setup was used. The experimental setup used, includes a white light source (HL–2000–LL, produced by Ocean Insight, Dunedin, FL, USA) and a spectrophotometer (FLAME-S-VIS-NIR-ES, produced by Ocean Insight, Orlando, FL, USA). In particular, the white light source is characterized by an emission range from 360 nm to 1700 nm, and the spectrophotometer has a detection range between 350 nm and 1000 nm. The two POF patches, having a diameter of 1 mm, were used to launch the light inside the waveguide core and to collect it at the output. The connection between the two POF patches and, respectively, the light source and the spectrophotometer was carried out using SMA connectors. Finally, the spectrophotometer was connected to a computer in order to acquire and process the experimental data. An actual image of the setup used is provided in Figure 5.

The optical characterization was carried out by testing the three configurations, previously presented, with several solutions at different refractive indices. In particular, six water–glycerin solutions were prepared by mixing water with different percentages of glycerol in order to obtain refractive indices between 1.332 RIU and 1.384 RIU. Before being used, each solution was checked using a commercial Abbe refractometer (RMI, Exacta + Optech GmbH, Munich, Germany).

## 4. Results and Discussion

### 4.1. Experimental Measurements

**Figure 6:**
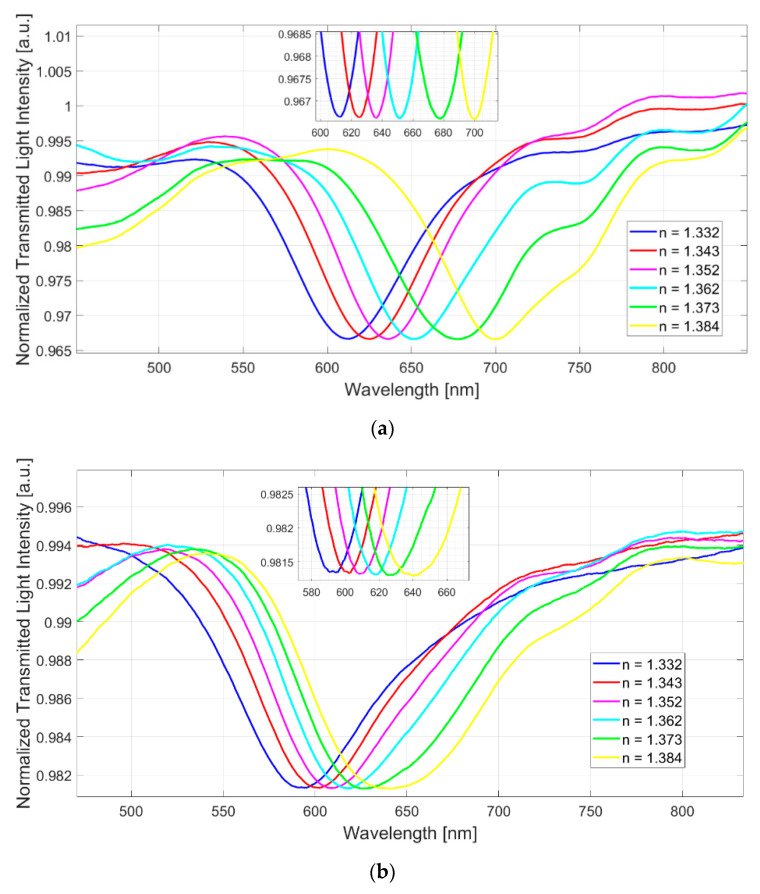

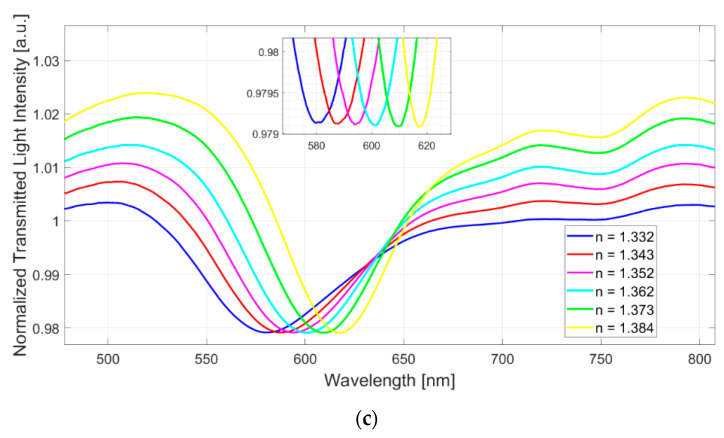
SPR spectra normalized to the reference spectrum (acquired in air) at different external refractive indices for (**a**) Configuration 1, (**b**) Configuration 2, and (**c**) Configuration 3.

Figure 6 shows, for all three configurations, the spectra obtained by carrying out a normalization on the spectrum acquired in air, i.e., without any solution on the plasmonic chip. In this instance, in fact, the necessary condition to satisfy the surface plasmon resonance is not fulfilled, hence it can be used as a reference spectrum for the normalization.

As can be seen from Figure 6, in each configuration, when the refractive index of the solution placed on the sensitive region of the sensor systems increases, a shift of the resonance wavelength to higher values can be observed (red shift).

In a similar way to the numerical results, when the core refractive index increases, at the same external refractive index range, the resonance wavelength range decreases (the SPR wavelengths shift on the left when the core refractive index increases, see Figure 1 and Figure 6).

**Figure 7:**
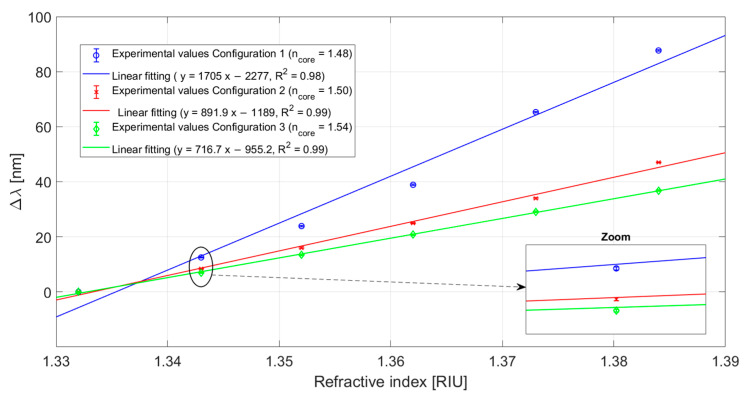
Variation in resonance wavelength (∆λ) as a function of the external refractive index. The linear fittings (solid lines) and error bars (zoom inset) of the experimental values are also reported for all the configurations.

In Figure 7 it is shown the resonance wavelengths versus refractive indices used to test the sensor system configurations. The experimental values obtained for each configuration were interpolated through a linear fitting in order to carry out a first order analysis. This kind of fitting appears to be suitable because of the R^2^ values equal to 0.98 for Configuration 1 and of 0.99 for Configuration 2 and Configuration 3. Each experimental value represents the mean of three successive measurements and the respective standard deviations (error bars) are shown in Figure 7 and are more evident in the zoom inset. The error bars were calculated as the maximum variation in resonance wavelength in the three subsequent measurements and they are equal to 0.2 nm.

The sensitivity, as indicated in Equation (3), can be calculated as the slope of the linear functions reported in Figure 7, and resulted equal to 1705 nm/RIU for Configuration 1, approximately equal to 892 nm/RIU for Configuration 2, and equal to 717 nm/RIU for Configuration 3. Furthermore, the resolution can be calculated as indicated in Equation (4), meaning as the reciprocal of the sensitivity multiplied by the measurement error (0.2 nm). So, the resolution, in terms of minimum detectable refractive index change, resulted in a value equal to 8.8 × 10^−4^ for Configuration 1, 1.68 × 10^−3^ for Configuration 2, and 2.09 × 10^−3^ for Configuration 3.

### 4.2. Comparative Analysis

The experimental results reported in Section 4.1 are summarized in Table 1. More specifically, as reported in Table 1, following the experimental results obtained, it is clear that the shift in resonance wavelength (δ*λ_res_*), for a fixed variation in the refractive index (δ*n_s_*), increases when the refractive index of the core decreases. Hence, the sensitivity increases as the refractive index of the core decreases, in a similar way to what obtained in [29]. As a consequence, it can be determined that the SPR curve width decreases with the increase in the fiber core refractive index, as shown in Figure 6. In particular, the SPR curve width changes for high solution’s refractive indices. This aspect is related to the fact that, concerning Configuration 2 and Configuration 3, a minor number of higher order modes, which are the more sensitive ones [27,28], is able to satisfy the SPR condition, with respect to Configuration 1. As a consequence, at the increasing of the core refractive index, the SPR spectra resulted narrower and the performance in terms of both sensitivity and resolution decreased with respect to Configuration 1.

Furthermore, it is important to stress that the performance obtained by Configuration 1, having a refractive index of the core equal to 1.48, are very similar to the one achieved by SPR platforms based on POFs having a core made of PMMA whose refractive index is equal to 1.49 [26].

Finally, Table 2 reports a comparative analysis, in terms of average sensitivity computed in the considered range for each configuration, between several polymer-based plasmonic sensors already presented in the literature. As shown in Table 2, the sensor’s performances of the best configuration (Configuration 1) are comparable with similar polymer-based sensors. However, we could improve the performance by changing the cross-section dimension/shape of the trench via an optimization analysis obtained by fixing the refractive indices. In fact, the proposed sensitive approach offers the possibility to achieve significant benefits by exploiting the capability of 3D printing techniques (e.g., the shape and the dimensions of the 3D-printed trench could be modified to change the sensor performance).

## 5. Conclusions

A comparative analysis between polymer-based plasmonic sensor chips was carried out by numerical and experimental results. The results indicated that better optical performances can be achieved by using an optical adhesive with a refractive index value of 1.48 RIU.

The obtained experimental results matched the simulations and can be used to develop the best SPR sensor configuration to implement disposable bio/chemical sensor chips via low-cost receptor layers, e.g., molecularly imprinted polymers and optimized plasmonic platforms based on simple 3D printed holders, optical adhesives, and sputtered gold nanofilms. Moreover, exploiting the proposed 3D-printing approach combined with POFs and optical adhesives, the optical waveguide used to excite the SPR phenomenon could be modified to improve the sensor performances. In a similar way, a useful shape can be designed (e.g., V-shape or U-shape geometry) for the optical waveguide in order to realize simple IoT sensor systems via a connection with a smartphone’s Light Emission Diode flash-light and a Charge Coupled Device.

## Figures and Tables

**Table 1:** Performance comparison of the three presented plasmonic configurations.

Configuration	$n_{\mathrm{core}}\;\left[\mathrm{RIU}\right]$	$S\;\left[\frac{\mathrm{nm}}{\mathrm{RIU}}\right]$	Δn [RIU]	$R^{2}$
Configuration 1	1.48	1705	0.88 × 10^−3^	0.98
Configuration 2	1.50	892	1.68 × 10^−3^	0.99
Configuration 3	1.54	717	2.09 × 10^−3^	0.99

**Table 2:** Performance comparison, in terms of average sensitivity in the considered range, of several SPR configurations based on polymeric waveguides.

Sensor Technology	$S\;\left[\frac{\mathrm{nm}}{\mathrm{RIU}}\right]$	Reference
NOA88 in 3D-printed VeroClear	710	[25]
D-shaped POF	~1800	[26]
Symmetrically etched POF	1600	[30]
U-shape tapered POF	1534.53	[31]
Polished POF	1174	[32]
V-grooves along the POF	1546	[33]
Side-polished macrobend POF	1233	[34]
Waveguide based on plastic optical fiber shell	1026	[35]
NOA148 in 3D-printed resin	1705	This work

## Data Availability

The data are available on reasonable request from the corresponding author.
